# Complexity, adaptations and variations in the secondary insemination system of female Dermanyssina mites (Acari: Anactinothrichida: Gamasida): the case of *Afrocypholaelaps africana*

**DOI:** 10.1007/s10493-017-0158-2

**Published:** 2017-07-27

**Authors:** A. Di Palma, O. D. Seeman, G. Alberti

**Affiliations:** 1 Department of the Science of Agriculture Food and Environment, University of Foggia, 71100 Foggia, Italy; 2 Queensland Museum, South Brisbane, Brisbane, Australia; 3 Zoologisches Institut und Museum, Universität Greifswald, 17489 Greifswald, Germany

**Keywords:** Sperm transfer, Podospermy, Sperm access system, Spermatheca, Female cryptic choice, Sacculus foemineus

## Abstract

Gamasine mites, mainly of the taxon Dermanyssina, possess a secondarily evolved insemination system (sperm access system), of which there are two, generally recognized, structurally different types, the laelapid- and the phytoseiid-type. The ultrastructure of the female sperm access system in *Afrocypholaelaps africana* is described. It consists of paired insemination pores, opening between the bases of legs three and four, and paired cuticle-lined tubules that converge into a large, sack-like spermatheca, remarkably cuticle-lined as well. The entire spermatheca and part of the tubules are embedded in a peculiar syncytial tissue where numerous sperm cells are present. The general organization of this insemination system is of the laelapid-type. However, it presents striking structural differences, compared with the systems described in *Varroa destructor* and *Hattena cometis*, the other gamasine mites having a laelapid-type system studied ultrastructurally until now. The functional morphology, complexity and variations of the sperm access system in Dermanyssina are discussed and correlated with the evolutionary biology of the group.

## Introduction

Mites are currently classified in two superorders, Anactinotrichida (=Parasitiformes) and Actinotrichida (=Acariformes) (Lindquist et al. 2009). Among Anactinotrichida gamasine mites represent the largest and most diverse group. Their reproductive behavior is not known in detail for most taxa. However, it seems that they all use some method of direct sperm transfer, with the male transferring sperm into the female body by way of either the female primary genital opening or, in some taxa, secondary insemination pores often located close to the leg coxae (Evans 1992; Alberti and Coons 1999; Krantz 2009). Such pores are connected to a complex system of tubes and reservoirs called the sperm access system, or secondary insemination system, where the sperm are received, stored and transferred to the ovary. Hence, the sperm access system is somehow associated with the female reproductive system, yet still separated from it.

Among Anactinotrichida, the sperm access system is known only in some groups of the order Mesostigmata, particularly the large subcohort Dermanyssina and the small family Heterozerconidae belonging to the cohort Heterozerconina (Evans 1992; Alberti and Coons 1999; Krantz 2009). In both cases, corresponding males are provided with a sperm transfer process (spermatodactyl) necessary to introduce the sperm into the insemination pores (Alberti 2002a, b; Di Palma and Alberti 2005; Di Palma et al. 2008). However, the sperm access system in Heterozerconidae is considered secondarily evolved, as shown by Di Palma et al. (2015), and is not discussed further here.

The sperm access system observed in Dermanyssina can be very complex but two basic, quite different types are known: the laelapid- and the phytoseiid-type (Michael 1892; Athias-Henriot 1968; Lee 1974; Evans and Till 1979; Evans 1992). According to earlier light microscopy (LM) studies, the laelapid-type consisted of paired insemination pores continuing into paired tubules meeting medially in an unpaired spermatheca, whereas the phytoseiid-type was thought to present two separate spermathecae connected to the ovary by means of separate routes (Fain 1963; Athias-Henriot 1971; Amano and Chant 1978; Evans and Till 1979). However, more recent detailed ultrastructural studies of the phytoseiid-type (Alberti 1988; Di Palma and Alberti 2001; Alberti and Di Palma 2002) showed that, like the laelapid-type ultrastructurally studied in *Varroa* (Alberti and Hänel 1986), it represented one continuous system whose spermathecae were medially connected by a belt-like tissue. This information was based on electron microscopical studies (EM), which are fundamental in clarifying the true organization of the anatomy and functional morphology of relatively small mites as Dermanyssina (usually 0.5–1.0 mm).

Moreover, in more recent studies on a species of Veigaiidae, a lower-derived dermanyssine taxon (Klompen et al. 2007), Alberti et al. (2009) described a sperm access system showing a certain similarity with the phytoseiid-type even though several differences were evident, so that it was difficult to simply classify it as a phytoseiid-type. Finally, Di Palma et al. (2013), in a more derived dermanyssine family (Ameroseiidae), described a sperm access system that, even though it lacked the extreme differences found in the veigaiid species, was considered as a variation of the laelapid-type.

Therefore, some steps must have been taken in the evolution of the complex secondary insemination system in Dermanyssina, and hence this structure is probably more diverse than the “classical” laelapid- and phytoseiid-types. In this respect, it is remarkable that several papers reporting light microscopical observations on the sperm access system in different families among Dermanyssina (e.g., Fain 1963; Athias-Henriot 1967; Lindquist 1995; Walter and Lindquist 1989, 1997), describe sperm access systems that, even though assigned to one type or the other (phytoseiid or laelapid), show different degrees of development of their components. These differences may represent different evolutionary steps from one sperm access system towards the other and have, of course, functional meaning as well; but to clarify this aspect, observations based on ultrastructural studies are needed.

In pursuit of this need, here we present studies of the sperm access system in *Afrocypholaelaps africana* (Evans), a flower-inhabiting species phoretic on insects, especially honeybees (Seeman and Walter 1995), and belonging to the family Ameroseiidae. This family has been assumed to have the laelapid type of sperm access system, but light microscopy observations suggest, in *A. africana*, an organization quite different from the “classical” laelapid type described in *Varroa destructor* Anderson and Trueman (Alberti and Hänel 1986) as well as from the one already described in *Hattena cometis* Domrow (Di Palma et al. 2013) belonging to the same family. Our contribution aims to improve our knowledge of this peculiar insemination system, the evolutionary biology of gamasine mites and their mechanism of sperm selection.

## Materials and methods

Females of *A. africana* were collected by O.D.S. ex flowers of River Mangrove, *Aegiceras corniculatum* at Fairfield, Brisbane, Queensland, Australia 27°30′19.7″S, 153°00′48.09″E in October 2012. Slide-mounted reference material from this collection is deposited in the Queensland Museum (QMS 107495-107500).

Females prepared for transmission electron microscopy (TEM) were dissected under a stereomicroscope in cold (ca. 4 °C) fixative, removing the anterior region of the body. After prefixation in 3.5% glutaraldehyde in phosphate buffer (pH 7.4; 0.1 M) at 4 °C for 3 h, the fixative was diluted with buffer (1:4) and the specimens were sent to Greifswald laboratory (Germany) where they were rinsed in buffer solution and subsequently post fixed in 2% OsO4-solution for 2 h. Hence they were dehydrated in graded ethanols and embedded in Spurr’s resin. Polymerization occurred at 60 °C. Ultrathin sectioning was performed with a Leica Ultracut UCT using Diatome diamond knives. The sections were double-stained with uranyl acetate and lead citrate (Reynolds 1963). Observations and micrographs were performed with a JEOL JEM-1011 transmission electron microscope.

Specimens for light microscopy (LM) were macerated in lactic acid, mounted on slides using Hoyer’s medium (Krantz and Walter 2009), and observed and photographed with an Olympus BX60 light microscope provided with an Axio Cam MRC Zeiss digital camera.

## Results

**Fig. 1:**
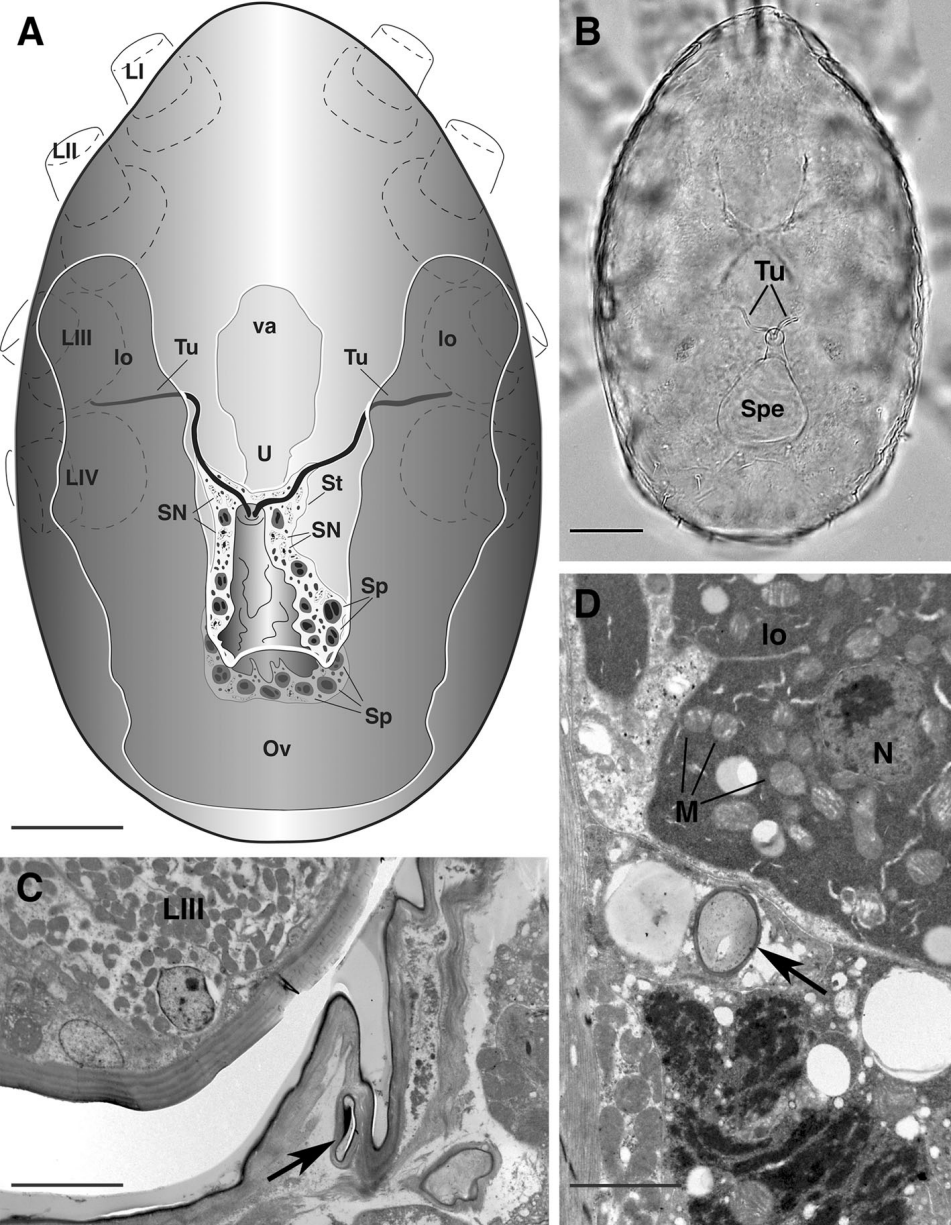
*Afrocypholaelaps africana*: female secondary insemination system. **a** Semi-schematic reconstruction, dorsal view. **b** LM, dorsal overview of the secondary insemination system: the two tubules entering the spermatheca are visible. **c** TEM, cross section at the level of the third pair of coxae showing the tubule on one side (*black arrow*). **d** TEM, cross section of the tubule (*black arrow*) more posteriorly in the body at the level of the lyrate organ of the reproductive system. LI-IV, leg I-IV; lo, lyrate organ; M, mitochondria; N, nucleus of the lyrate organ; Ov, ovary; Sp, sperm cell; SN, somatic nucleus of the syncytial tissue; Spe, spermatheca; St, syncytial tissue; Tu, tubule; U, uterus, va, vagina. *Scale bar* 50 µm (**a**, **b**); 2 µm (**c**, **d**)

The female sperm access system in *A. africana* is located in the posterior region of the body and readily visible in light microscopy slides (Fig. 1a, b). It starts with paired insemination pores located between the third and fourth pair of leg coxae and is composed of paired tubuli converging into a large sack-like spermatheca (Fig. 1a, b).

**Fig. 2:**
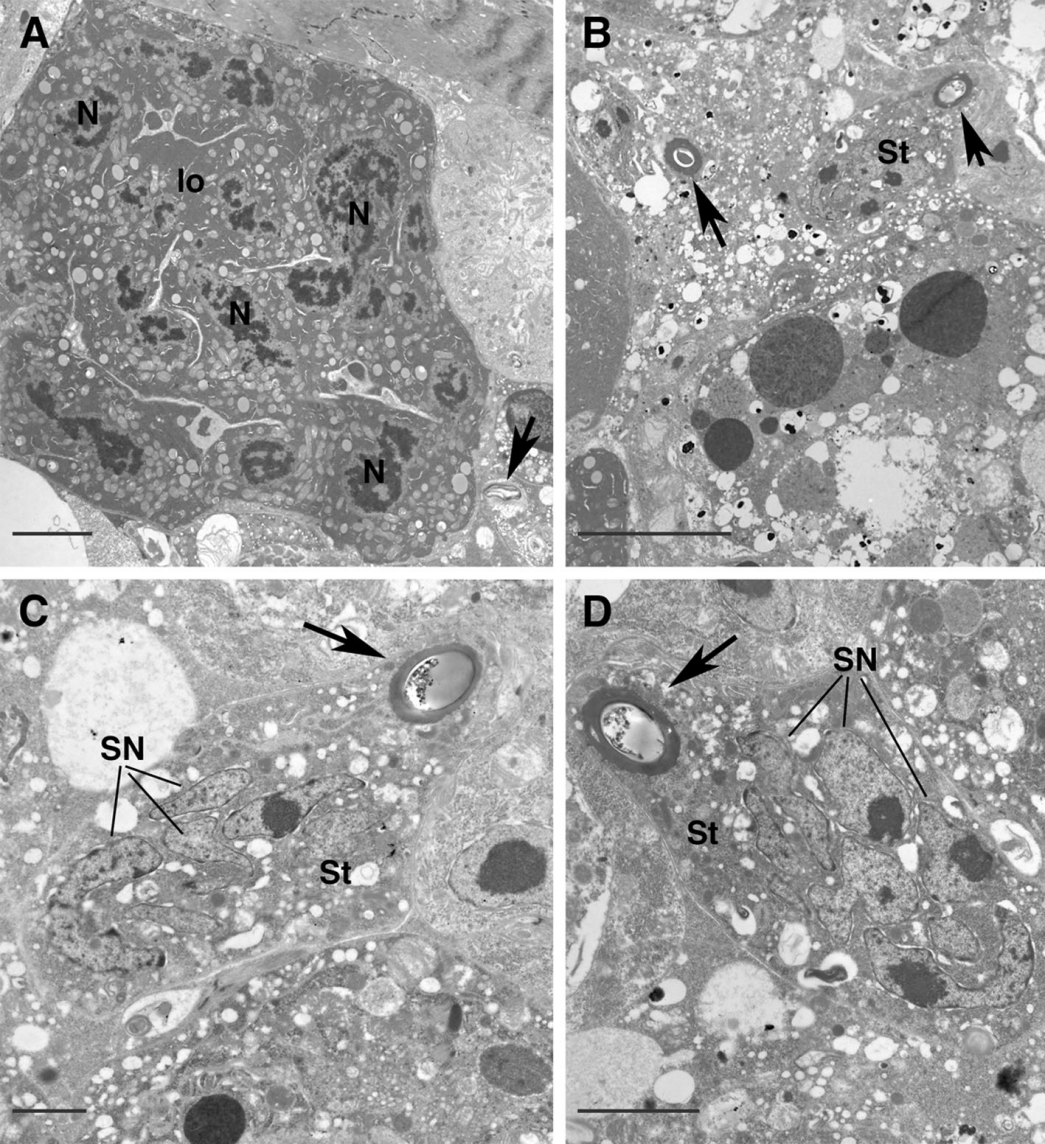
*Afrocypholaelaps africana*: female secondary insemination system, TEM cross sections. **a** The tubule (*black arrow*) from one side ventrally located to the lyrate organ of the reproductive system. **b** Cross section more posteriorly into the body: overview of the two tubules (*arrows*) getting closer and surrounded by the syncytial tissue. **c**, **d** Detail of the tubules (*black arrows*) on both sides embedded by the syncytial tissue. lo, lyrate organ; N, nucleus of the lyrate organ; SN, somatic nucleus of the syncytial tissue; St, syncytial tissue. *Scale bar* 5 µm (**a**); 10 µm (**b**); 2 µm (**c**, **d**)

The insemination pores (solenostomes) are small pores, close to coxae III (Fig. 1c), and continue as cuticle-lined tubules inside the body, one on each side. The lumen of these tubules is sometimes flattened (Fig. 1c) and bordered by a multilayered cuticle with a thin more electron-dense inner layer. The tubules run inside the body posteromedially and dorsally directed (Fig. 1a, b), close to the lyrate organ of the female reproductive system (Figs. 1d, 2a). The lyrate organ represents the nutritive part of the gonad, mainly composed of a syncytial nutritive tissue connected to the oocytes by nutritive cords (Evans 1992; Alberti and Hänel 1986; Alberti 1988; Alberti and Coons 1999; Di Palma and Alberti 2001) (Figs. 1d, 2a).

**Fig. 3:**
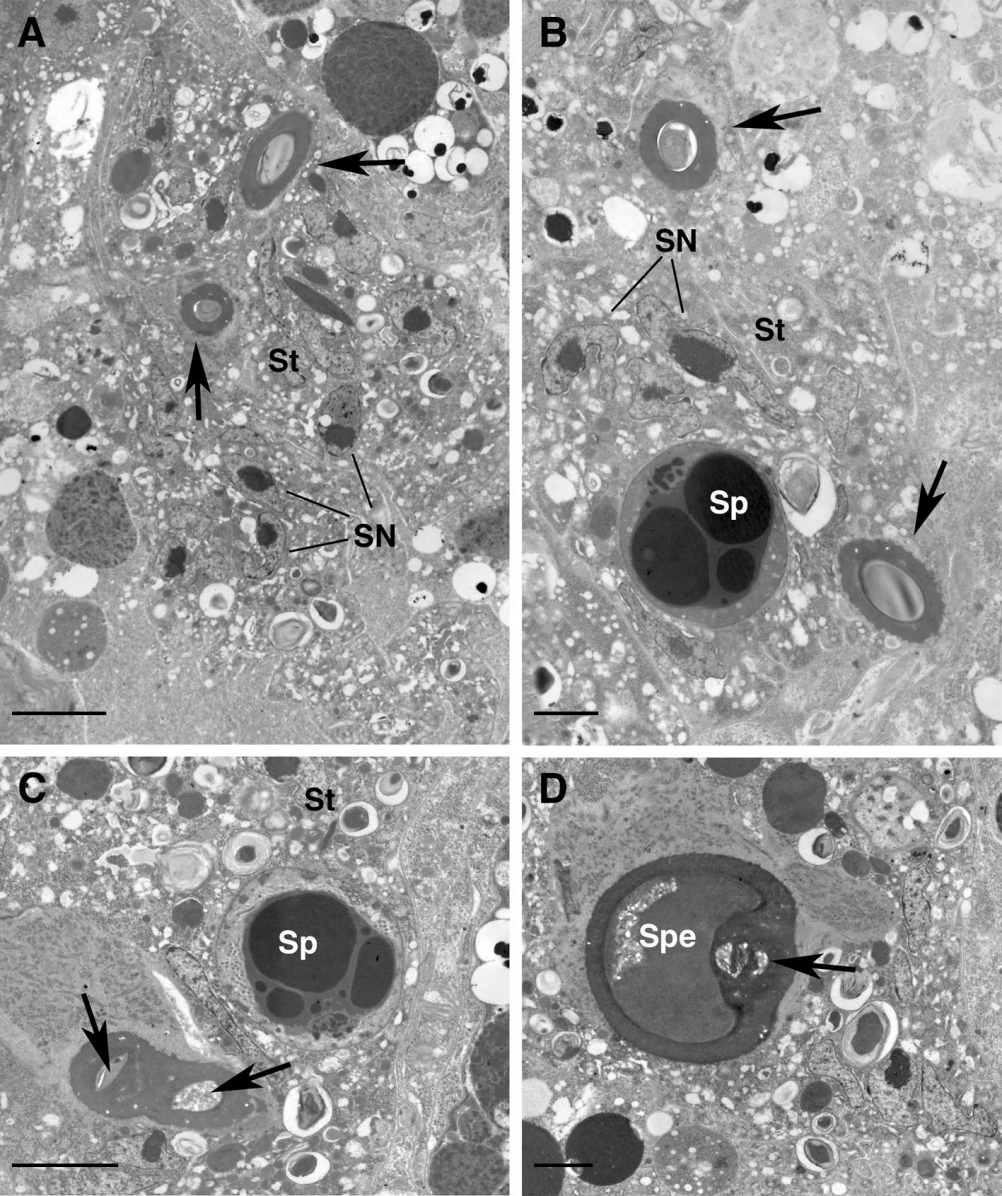
*Afrocypholaelaps africana*: female secondary insemination system, TEM cross sections. **a** Tubules coming from each side of the body and getting closer (*black arrows*). Both are embedded into the syncytial tissue. **b** Detail of the two tubules (*black arrows*) embedded into the syncytial tissue where sperm cells, beside the somatic nuclei, are present. **c** Detail of the tubules so closed that the cuticle is fused but it is still possible to distinguish the two lumens (*black arrows*). **d** Detail of the tubules entering into the round-shaped spermatheca. At this level the lumens of the two tubules are fused (*black arrow*). SN, somatic nucleus of the syncytial tissue; Sp, sperm cell; Spe, spermatheca; St, syncytial tissue. *Scale bar* 5 µm (**a**); 2 µm (**b**–**d**)

**Fig. 4:**
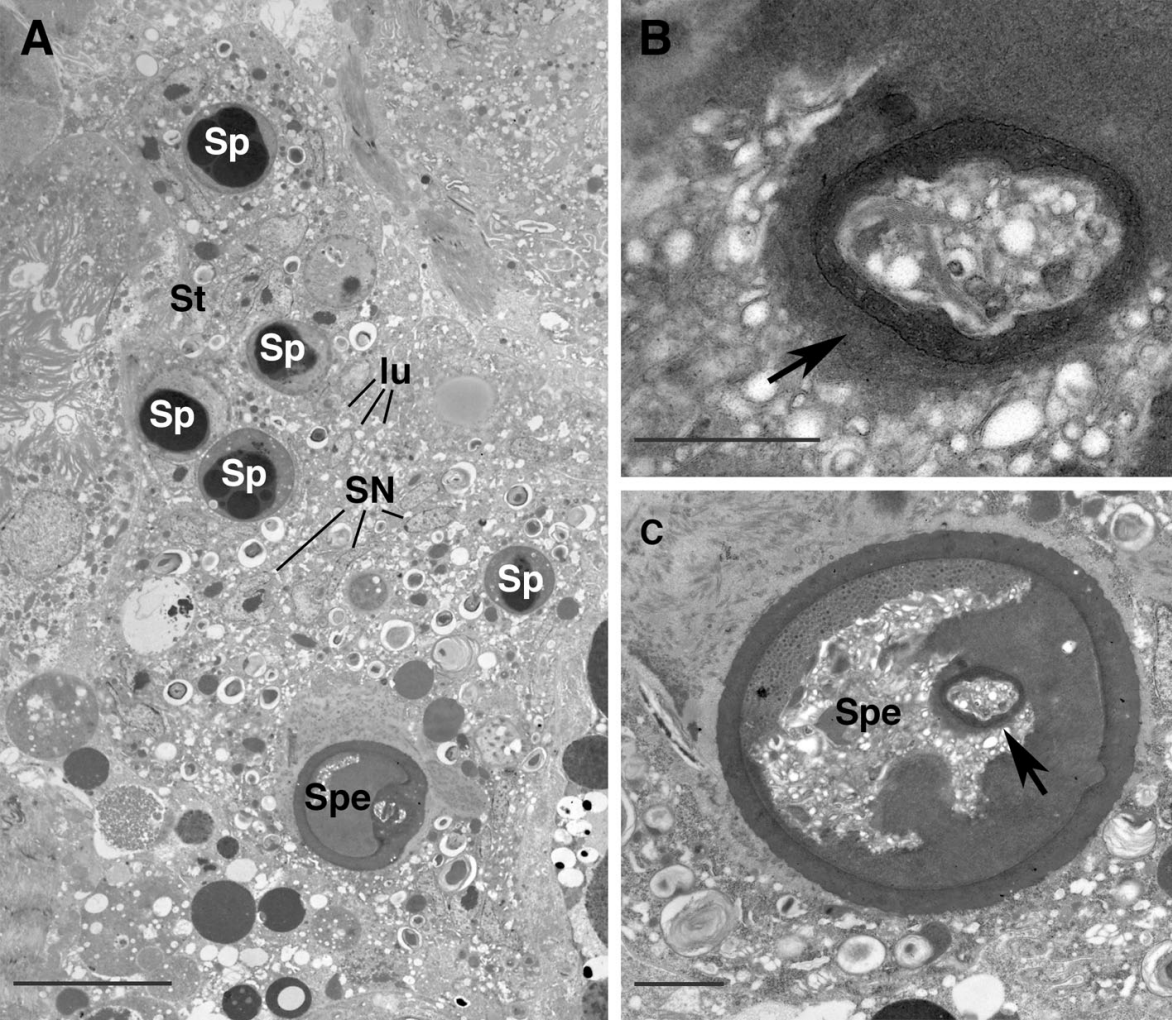
*Afrocypholaelaps africana*: female secondary insemination system, TEM cross sections. **a** Overview of the spermatheca at the anterior region embedded in the syncytial tissue full of sperm cells, electron lucent vesicles and somatic nuclei. **b** Detail of the lumen (*black arrow*) of the two fused tubules entering the spermatheca. **c** Detail of the spermatheca at the anterior level: it appears roundish in shape with the common lumen of the two tubules (*arrow*). lu, electron lucent vesicle; SN, somatic nucleus of the syncytial tissue; Sp, sperm cell; St, syncytial tissue; Spe, spermatheca. *Scale bar* 10 µm (**a**); 1 µm (**b**); 2 µm (**c**)

At the level of the lyrate organ, the tubules show a thinner cuticle and some material of unknown structure is visible in the lumen (Figs. 1d, 2a); more posteriorly, the tubules become narrower in cross section (Figs. 1a, b, 2b) and show a thicker cuticle (although the thickness of the cuticle might be due to orientation of the sections) (Fig. 2b); moreover, they are embedded in an electron lucent tissue (Fig. 2b–d). This tissue is a syncytium with packed irregularly shaped nuclei provided with scattered chromatin or roundish nucleoli and a cytoplasm that is rich in free ribosomes, electron-lucent vesicles and a few droplets of dark secretions (Fig. 2c, d). Even more posteriorly, the tubules become very close while the syncytium becomes larger (Fig. 3a) and some sperm cells are evident in the syncytium (Fig. 3b). Finally the two ducts join into one common lumen (Figs. 3c, 4a–c arrows) that continues into a wide, still cuticle-lined structure, with a roundish shape in cross section: the spermatheca or sacculus foemineus (Figs. 1a, b, 3d). This structure is completely embedded in the syncytium where, beside nuclei, ribosomes, droplets of secretions and electron lucent vesicles, large sperm become more abundant (Fig. 4a). Remarkably, no sperm cells have been observed in the tubules whose lumen looks smaller than the sperm cells themselves (Fig. 3b, c).

**Fig. 5:**
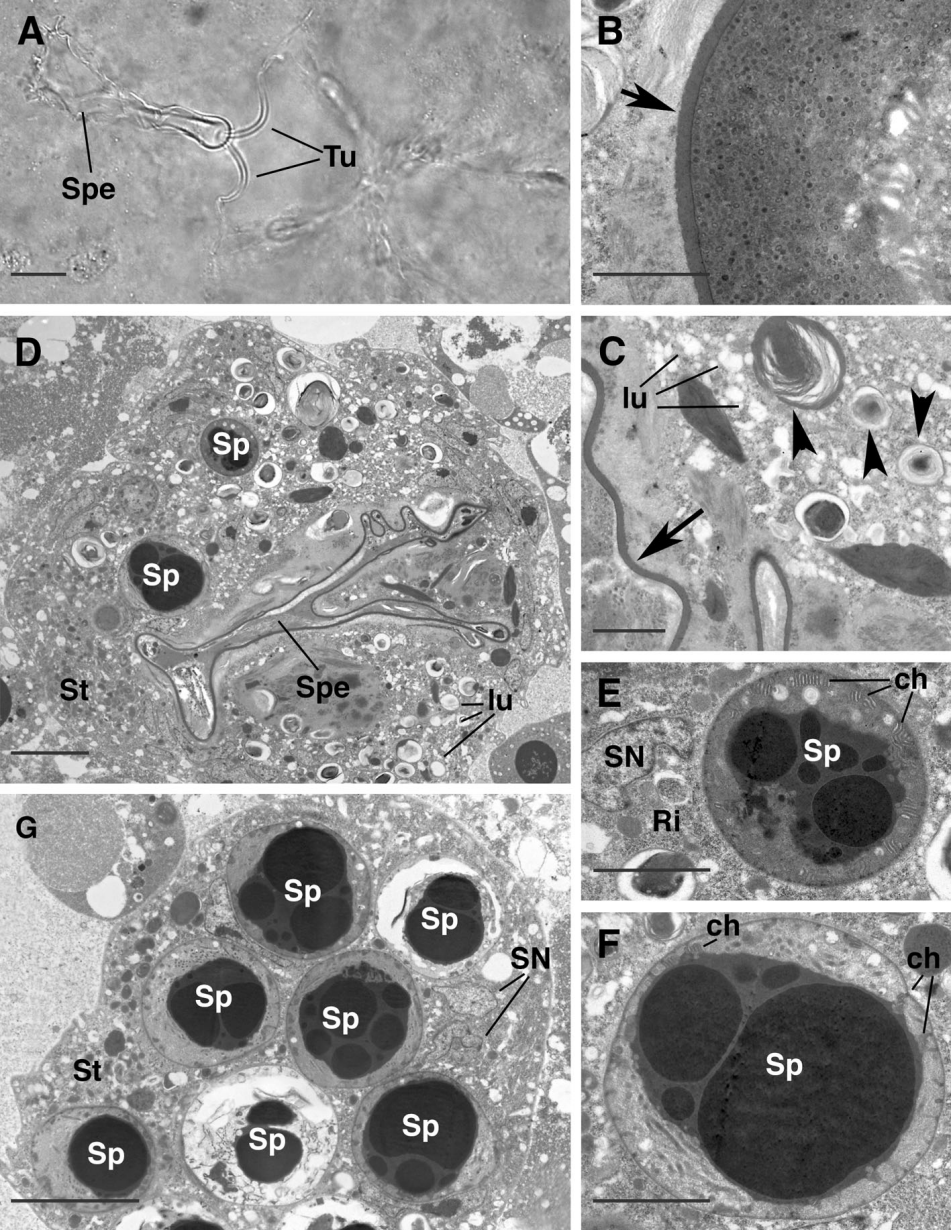
*Afrocypholaelaps africana*: female secondary insemination system. **a** LM, dorsal view of the secondary insemination system: the two tubules are evident entering the spermatheca that, in this specimen, shows folded walls. **b** TEM cross section: detail of the cuticle lined wall of the spermatheca (*arrow*). **c** TEM cross section: detail of the spermatheca in a more posterior region: the thin cuticle wall (*arrow*), unstructured material (*arrow heads*) and electron lucent vesicles are evident. **d** TEM cross section: overview of the spermatheca in a more posterior region. It appears with folded wall and completely embedded in the syncytial tissue where numerous sperm cells and electron lucent vesicles are evident. Remarkably no cells (either somatic or sperm) are visible in the lumen of the spermatheca. **e** TEM cross section: detail of a sperm cell embedded in the syncytial tissue. Numerous small flat peripheral chambers are evident close to the cell membrane while most of the cytoplasm is occupied by electron-dense droplets of cromathin. **f** TEM cross section: detail of a sperm cell: in this cell the peripheral chamber are less numerous and evident since they are under formation. **g** TEM cross section in the posterior region of the body where the spermatheca is not present anymore but the syncytial tissue embedding the sperm cells is still visible. ch, peripheral chamber; lu, electron lucent vesicle; Ri, ribosomes; SN, somatic nucleus of the syncytial tissue; Sp, sperm cell; Spe, spermatheca; St, syncytial tissue; Tu, tubule. *Scale bar* 20 µm (**a**); 2 µm (**b**, **c**, **e**, **f**) 5 µm (**d**, **g**)

The spermatheca, the roundish-shaped structure where the tubules enter joined into a common lumen (Fig. 4a–c), shows a uniform cuticle (Fig. 4a–c) anteriorly while, more posteriorly, it enlarges into a sack-like structure (Fig. 1a, b) whose walls may be sometimes folded (Fig. 5a, d) and with a thinner cuticle (Fig. 5b, c). Inside this large sack-like structure some unstructured material is visible, as are several electron lucent vesicles (Fig. 5b, c), while the whole sack is embedded into the electron lucent syncytium observed already at the level of the two tubules (Fig. 5d). The spermatheca presents a thin layer of cuticle where no obvious opening was detected; moreover, no sperm cells were ever observed inside the spermatheca, but only in the syncytial tissue surrounding it (Figs. 4a, 5d).

In the syncytium, the number of sperm cells, electron lucent vesicles, and the amount of unstructured material, is higher in the posterior region of the spermatheca than the anterior (Figs. 1a, 5d). Moreover, in closer view, a larger number of ribosomes are evident posteriorly as well (Fig. 5e).

The sperm cells observed in the syncytium are considered to represent spermatozoa under the capacitation process. They are ovoid or spherical in shape, some with evident elongated flat peripheral chambers close to the cell membrane (Fig. 5e) while in others such chambers are less numerous or evident because they are still being formed (Fig. 5f). Moreover the cytoplasm is devoid of most of the cellular organelles, while very large electron-dense droplets of chromatin occupy almost the entire space (Fig. 5e–g).

The electron-lucent syncytium containing the sperm cells extends laterally, ventrally and posteriorly to the spermatheca (Fig. 5d, g) and is located close to the ventral uterus and the dorsal lyrate organ of the female reproductive system (Fig. 1a).

## Discussion

According to our observations, the sperm access system in female *A. africana* presents a pair of insemination pores (solenostomes), located between the third and fourth pair of legs, and paired cuticle-lined tubules which come together, side-by-side, to merge into one duct leading into the enormous, cuticle-lined sack-like reservoir (spermatheca/sacculus foemineus) (Figs. 1a, 2b, 3c, d, 4b, c). Hence, the general organization of this sperm access system resembles the laelapid-type (Michael 1892; Fain 1963; Athias-Henriot 1967; Alberti and Hänel 1986; Di Palma et al. 2013) as anticipated according to the systematic position of the species: Gamasida: Dermanyssina: Ameroseiidae.

Regarding the functional morphology of the laelapid sperm access system, it is obvious, according to behavioral observations (Michael 1892; Young 1968), that males insert the spermatodactyl into the insemination pores and release the sperm cells. Then the spermatozoa move through the tubules to reach the reservoir (spermatheca/sacculus foemineus) where they are stored until they move to the ovary (Alberti and Hänel 1986; Di Palma et al. 2013).

In our sections no sperm cells were ever detected inside the tubules, whose cross section is smaller than the diameter of the sperm cells (see Fig. 3b, c). Therefore, sperm cells must either alter their form to pass through the ducts, or the tubules can become enlarged, as reported in *Varroa* by Alberti and Hänel (1986), although none of our sections showed enlargement of the ducts.

The spermatheca may present different shapes: in some slide-mounted specimens it appears sack-like (Fig. 1b), while in others it shows folded walls (Fig. 5a). One might assume that the spermatheca is enlarged when full of sperm cells, while it shrinks when vacated of sperm. Yet, we never observed sperm in the large spermatheca, while numerous spermatozoa were observed in the syncytium surrounding the two tubules and completely embedding the spermatheca (Figs. 3b, c, 4a). Of course, this might be due to the sperm having moved already from the spermatheca to the syncytium. In any case, no obvious opening was evident in the cuticle-lined walls of the spermatheca. So, even if we assume that the sperm released by the male into the insemination pores can squeeze themselves through the tubules and reach the spermatheca, it is still an open question how they can get out of it and reach the syncytium. Regarding the ability of the spermatozoa to get out of a cuticle-lined spermatheca devoid of any obvious opening, we might assume that the sperm cells are somehow able to penetrate the cuticular wall; in this respect, the cuticle of the spermatheca is thinner than that observed in the tubules (Fig. 5c, d). This possibility has already been suggested by Di Palma and Alberti (2001) in phytoseiid mites and by Alberti et al. (2009) in veigaiid mites. In both cases, the manner by which the sperm emerged from the cuticle-lined vesicles and reached the ovary was unclear, and the possibility that the spermatozoa can penetrate through a thin cuticular region was proposed. Moreover, in an entirely unrelated taxon, the spider mites (Tetranychidae, Actinotrichida), sperm is reported to penetrate the specialized wall of the receptacle (Alberti and Storch 1976).

Hence, somehow the sperm cells reach the syncytium and from there, due to an active penetration of the female tissues, as suggested by Alberti and Hänel (1986), reach the ovary, where they were observed. In support of this idea, the number of sperm cells is higher in the posterior region of the syncytium, where it is closest to the ovary.

Regarding some peculiarities of the sperm access system in *A. africana*, all the components are cuticle lined, so the entire system is clearly visible under light microscopy (Figs. 1b, 5a). This apparentness is not always evident for the laelapid-type of sperm access system, as indicated by some light microscopy studies (i.e. Athias-Henriot 1967), where the spermatheca/sacculus foemineus is sometimes difficult to discern. This was confirmed by ultrastructural studies of *Varroa* (Alberti and Hänel 1986) and *Hattena* (Di Palma et al. 2013) where the spermatheca/sacculus foemineus has walls consisting of cells arranged in a syncytium but without a cuticle layer (that is instead present in *Afrocypholaelaps*). So far *A. africana* is the only ultrastructurally studied species with a laelapid-type of sperm access system having a cuticle-lined spermatheca; this explains why the structure is obvious under light microscopy while in *Varroa* and *Hattena* is difficult to detect in macerated specimens mounted on slides.

*Afrocypholaelaps*, *Hattena* and *Varroa* all share similarities in the general organization of the sperm access system (i.e., insemination pores, access ducts connected to a common spermatheca), so that they all belong to the laelapid-type. However, in *Varroa* the sperm access system is much more differentiated, with ultrastructurally discernible tubules, rami and a sperm duct leading to the spermatheca; while in *Hattena* the organization of the system appears simpler (no division in tubules and rami and no sperm duct), with a reduction of cuticular parts (very short access ducts, representing the only cuticle-lined components). *Afrocypholaelaps* and *Hattena* share some similarities, in that there is no division of the access ducts in ultrastructurally discernable tubules and rami, and no sperm duct is present. However, unlike *Hattena,* the tubules are much longer and all the components are cuticle-lined, including the spermatheca.

The differences between *Afrocypholaelaps* and *Hattena* are more remarkable because they belong to the same family. Along with *Neocypholaelaps*, they also share a similar way of life, living within flowers and moving between them on floral visitors such as insects and birds (Seeman and Walter 1995; Seeman 1996; Kar et al. 2015). In some regards, *Afrocypholaelaps* and *Hattena* seem more closely related to each other than many other members of the Ameroseiidae, as they share the loss of ambulacral claws and females have fine, smooth dorsal setae (Halliday 1997). However, the much reduced leg chaetotaxy of *A. africana* indicates that this species is a highly specialized ameroseiid mite: members of *Hattena* and *Neocypholaelaps* share a very similar leg chaetotaxy with their soil and foliar-dwelling kin such as *Ameroseius* (Evans 1963; Moraza 2006; de Moraes and Narita 2010; pers. obs.), but *A. africana* have lost many leg setae, particularly on leg II and III (Elsen 1972; pers. obs.). Thus, the surprisingly unique aspects of the sperm access system of *Afrocypholaelaps* are reflected in its morphology.

In all three species ultrastructurally studied (*Varroa*, *Hattena* and *Afrocypholaelaps*), there is a syncytium where the sperm cells are observed and that likely represent a kind of path to lead them from the spermatheca to the ovary, confirming an active penetration of the female tissues by the sperm. Yet it is intriguing that in *Afrocypholaelaps*, the spermatheca (where the spermatozoa are supposed to be stored) is cuticle lined, while in the other two cases, *Varroa* and *Hattena*, the spermatheca has no cuticular intima but is represented by a sack-like structure whose walls consist of a syncytium. In this respect the large cuticle-lined spermatheca observed in *Afrocypholaelaps* is ultrastructurally and functionally quite different from the other two cases; somehow, the sperm must find their way out of a cuticular wall where no opening has been detected.

One can think of a selection mode among spermatozoa or of a mechanism to discriminate among males connected to an ability of the sperm to get through a thin cuticular region and reach the surrounding syncytium and then the ovary. In this respect the female sperm access system in Dermanyssina perhaps represents a case of cryptic female choice: females can selectively favour one male’s chances of paternity over those of another, a phenomenon already known among insects and arachnids (Eberhard 1985). Alberti (2002a) hypothesised that, in dermanyssine mites, the complexity of both laelapid- and phytoseiid-type sperm access systems could therefore be a result of the interplay between females acquiring post-insemination sperm selection and males circumventing such defenses. His notion may be supported by the “difficult” and still obscure route that spermatozoa have to follow to emerge from the reservoirs in phytoseiids (Di Palma and Alberti 2001). Perhaps, in the case of *Afrocypholaelaps*, the cuticular walls of the spermatheca may “filter” sperm from wanted and unwanted copulations. In such cases, the female specifies the “rules of the game”, determining that some variants of the male morphology or behaviour will be more successful than others (e.g. in several spiders it has seemed advantageous for females to increase the difficulty of either sperm cell entry into or exiting from the spermatheca) (Eberhard 1985).

There are few examples of ultrastructural studies on the sperm access systems Dermanyssina, yet the occurrence of several variations in this system (Alberti and Hänel 1986; Di Palma and Alberti 2001; Alberti et al. 2009; Di Palma et al. 2013) demonstrates the existence of a complicated evolutionary trend or branching trends of the component structures. These studies provide further reason to urge additional observations of such systems in other gamasine families, even in species of several genera among the same family, to try to understand the evolutionary steps from one access system towards the other, and the variation within each major system. The latter plea is emphasized here by considering the differences between the taxonomically and ecologically similar genera *Hattena* and *Afrocypholaelaps*, and we note that the sperm access systems of other ameroseiid genera like *Ameroseius* and *Kleemannia*, living in very different habitats, would be useful subjects for future comparison. Our observations also urge similar such studies among a comparable variety of taxa manifesting the phytoseiid-type system, particularly among genera of the blattisociid subfamilies Platyseiinae and Blattisociinae (especially the highly diverse and speciose genus *Lasioseius*), to better understand the remarkable biological diffusion that Dermanyssina mites have had.
